# The Influence of Relationships on Engagement in an Australian Construction Industry Suicide Prevention Programme

**DOI:** 10.1002/ajim.70034

**Published:** 2025-10-31

**Authors:** Jorgen Gullestrup, Samantha Thomas, Tania King, Anthony D. LaMontagne

**Affiliations:** ^1^ Faculty of Health/School of Health and Social Development/Institute for Health Transformation Deakin University Geelong Victoria Australia; ^2^ Public Health, Faculty of Health/School of Health and Social Development/Institute for Health Transformation Deakin University Geelong Victoria Australia; ^3^ School of Global, Urban and Social Studies RMIT University Melbourne Victoria Australia

**Keywords:** gatekeeper training, help‐offering, peer led, suicide prevention, workplace

## Abstract

**Background:**

Every 45 seconds a person dies by suicide. Significantly more men than women die by suicide and most are of working age. Some industries such as the construction industry have higher rates of suicide. *MATES in Construction* is an Australian programme established in 2008 to reduce suicide rates in the construction industry.

The purpose of this study was to gain insight into the role‐relationships between participants, staff and the MATES in Construction (MATES) organisation in an industry‐based suicide prevention programme in the Australian construction industry.

**Methods:**

Semi‐structured qualitative interviews were conducted with 28 MATES programme volunteers. The data was analysed using a reflexive thematic analysis approach.

**Results:**

From the data two themes were constructed. Firstly, MATES staff relatability builds worker trust and facilitates engagement in the programme. As peer workers, MATES staff were trusted and modelled the desired outcomes of the programme. Secondly, the authentic industry base of the programme enhances worker trust and engagement. MATES could be trusted because it was part of the construction industry as an industry‐owned and ‐led organisation supported by both employers and unions. Union endorsement was seen as particularly important by workers.

**Conclusions:**

The relatability of MATES staff and MATES' industry base were seen as important drivers of worker engagement and participation in the programme. This study provides new insights into the importance of peer roles, authenticity, connection, and positionality of public health interventions as facilitators of engagement and participation in community‐based preventive interventions.

## 1 Introduction

Worldwide, suicide is a major public health issue and every 45 s a person dies by suicide, with more than 700,000 lives lost each year [1]. Globally suicide rates are 2.3 times higher for men than for women and more than 3 times higher in high‐income countries [1]. Amongst employed men, suicide rates tend to be higher amongst blue‐collar workers, and in lower‐skilled and less tertiary‐educated occupations [2, 3, 4].

Australian construction workers have been found to have suicide rates roughly twice the rate of other employed men [5] and while American construction workers account for only 7.4% of the US workforce the industry account for 17.9% of all suicides [6] a phenomenon also observed in other countries globally [7, 8]. The high rates of suicide are the result of a combination of personal‐ and industry‐related risk factors such as unmanaged psychosocial hazards [9, 10, 11]. The work‐relatedness of suicide often goes unrecorded and is rarely recorded as an injury‐at‐work [12]; however, research suggests that at least 10‐13% of all suicides have a level of work‐relatedness [13].

The World Health Organisation (WHO) has established the LIVE LIFE initiative and an implementation guide for suicide prevention in countries to support the objective of reducing the global suicide rate by one‐third before 2030 [14]. The WHO LIVE LIFE guide recommends a systematic approach including situational analysis, multisectoral collaboration, awareness‐raising and advocacy, capacity building, funding, surveillance and monitoring/evaluation. As an example of best‐practice community capacity building, the guide features the Australian workplace suicide prevention programme *MATES in Construction* (hereinafter MATES) [14].

MATES is a workplace mental health and suicide prevention programme, introduced to the Queensland construction industry in 2007 in response to significantly elevated suicide rates in the sector [15, 16]. The programme has since expanded to several other states in Australia as well as to New Zealand. The MATES programme has also established related programmes in the Mining, Energy, and Manufacturing industries [17]. Since inception MATES has trained more than 478,000 workers in the programme.

MATES programme has been extensively described elsewhere [18, 19, 20, 21]. In brief, MATES is an industry‐wide programme implemented at a worksite level by MATES‐employed Field Officers. These Field Officers support an extensive network of on‐site volunteer construction workers who agree to train and act as peer supports for their workmates. Field Officers engage with, and train, workers on sites at the following levels:

- **General Awareness Training** (GAT) for all workers on site – GAT is a 1‐h session using an ‘anger‐hope‐action’ engagement strategy to motivate workers to participate in suicide prevention on their worksite. Workers participating in GAT get a white hard hat sticker [21].
- **Connector Training** is the first‐level volunteer training in the MATES programme—the programme is delivered on site over 4 h based on LivingWorks safeTALK training. Connectors are volunteers agreeing to connect workmates to support if they are struggling with mental health or suicidality. MATES targets one Connector for every 20 workers on site at varying peer levels to optimise accessibility. Workers trained as Connectors get a green hard hat sticker.
- **ASIST Training** is the second level of volunteers trained by the MATES programme. LivingWorks ASIST is a 16‐h training course delivered to one or more key workers on site to be a resource to assist workers in acute distress referred from workers generally and site Connectors. ASIST workers get a blue hard hat sticker.

Trained volunteer Connectors and ASIST workers are encouraged to network with other volunteers on site to implement the MATES programme on their worksite in a manner suitable for that site. This is intended to create an on‐site network of suicide safety supported by a 24/7 support line and a MATES case management service for workers.

Evaluation of the MATES programme has found evidence of the effectiveness of the programme in improving mental health and suicide prevention literacy, reducing stigma, and improving helping intentions and behaviours leading to increased demand for case management support [18, 22, 23]. In addition, descriptive surveillance studies have shown a steady decline in male suicide rates in the construction sector compared to other working males in Australia, suggesting a possible contribution to this decline by the MATES programme [5, 24].

Previous studies have examined how the connection between construction workers, their social identity, mateship, and lived experience have motivated engagement in the MATES in Construction programme [21] and how networks of safety and social networks across an industry can provide a context that may shift social norms towards a more supportive and helping culture [19]. The current study builds on these and seeks to increase understanding of the relationships between construction workers and the MATES organisation, programme, and staff and how these relationships support the programme's strong uptake across the construction industry. This study seeks to investigate the following research question:

*What role do the relationships between construction workers and the MATES staff and organisation play in engagement in the MATES programme?*

## 2 Methodology

### 2.1 Approach

This study takes a ‘Big Q’ interpretivist approach to data analysis acknowledging that knowledge production inevitably is “partial, situated and contextual” [25]. A ‘Big Q’ interpretivist approach accepts that meaning is a subjective construct informed by both the researchers' subjectivity and participants' own ‘subjective experiences and sense‐making’ [26]. Using Reflexive Thematic Analysis, the authors actively participated in meaning generation from the data [25, 26, 27].

The approach to the data was informed by the positionality of the research team. Author 1 is a former construction worker and union leader as well as a founding CEO of the MATES programme. Author 1 also identifies as having lived and living experience of suicide and mental health crisis. This positionality of Author 1 facilitated rapport with study subjects during interviews. Author 2 has a background and expertise as a public health sociologist and specific experience in mental health and addiction research. Authors 3 and Author 4 have expertise in male‐dominated workplaces, occupational health & safety, workplace interventions, and suicide prevention.

Throughout the study process the research team met regularly on‐line and discussed the meaning created from the data. A reflexive approach was taken throughout the process where the positionality of the team was tested against assumptions, choices and thoughts generated from the data.

The study was approved by the Deakin University Human Research Ethics Committee.

### 2.2 Criteria for Recruitment

This study used a combination of convenience and purposive sampling. Recruited participants were individuals actively engaged in the MATES programme within the Australian state of Queensland, because this is the state where the programme has operated the longest and has had the widest diffusion within the industry. Study recruitment was in three stages. Initially six individuals were recruited through invitation by MATES staff to ensure diversity of workplace roles. These participants included workers, managers, industry leaders, policymakers and researchers. The second stage recruited nine participants from worksites following promotion of the study by MATES staff, posters and flyers on the jobs. The third stage specifically targeted recruitment of blue‐collar workers due to an over‐representation of white‐collar and management roles in the first two stages of interviews; this was done through promotion at MATES training events.

Participants registered an interest on‐line via a QR code. They were further required to complete appropriate consent forms before interviews. All participation in the study was voluntary and no incentives or compensation was offered by the research team or the MATES organisation.

### 2.3 Data Collection

Author 1 conducted all interviews except for one interview where Author 2 also participated to inform discussion about any need for changes to of the interview guide. Minor changes to the interview guide were made and approved by the ethics committee. All data was collected between January 2022 and February 2024. All interviews were video recorded with participant consent. Interviews were semi‐structured and supported by an interview guide (available in the Supporting material 1). All interviews were transcribed before data analysis. The duration of interviews was on average 49 min (range of 31–77 min). Participants were offered the transcripts for review following interviews, but no participants provided corrections or further comments after reviewing their transcripts.

### 2.4 Data Analysis

This study applied Braun and Clarke's six step Reflexive Thematic Analysis (RTA) approach [26]. These six steps are not applied sequentially or in a linear process but in an iterative manner moving backwards and forwards between steps as the research team discussed and worked with the data in a reflexive manner until the team could agree on a shared understanding of the data. The six steps were:

- **Step 1 –** Author 1 familiarised himself with the data by reviewing all transcripts in detail. During this process co‐authors also had access to the transcripts.
- **Step 2 –** Author 1 applied initial codes capturing both literal and latent meaning in the data.
- **Steps 3 and 4 –** Following reflection on the initial codes Author 1 refined initial codes and developed themes which were discussed by the research team until consensus was reached.
- **Step 5 –** Author 1 summarised the discussion and developed short descriptions of each theme.
- **Step 6 –** The writing up of the findings was done in an iterative manner, with Author 1 leading the writing in close consultation with the full research team.

## 3 Findings

Most of the 28 interview participants identified as having lived or living experience of mental ill‐health and/or suicidality (*n* = 26), most participants identified as male (*n* = 26) and were aged between 29 and 57 years (mean = 45 years). Participants performed a variety of roles in the industry from senior site management, union delegates, tradespersons, apprentices, labourers, and industry leaders playing a variety of roles in the MATES programme including Connectors and ASIST workers, facilitators of the programme on site, MATES Field Officers and a MATES Board member. Participants' work experience in the industry varied between four and 42 years (mean = 22 years).

Two themes were constructed from the data: (1) ‘MATES staff relatability builds worker trust and facilitates engagement in the programme’; and (2) ‘the authentic industry base of the programme enhances worker trust and engagement’. An overview of key data considered in the construction of these themes can be found as Supporting Material to this article.

Theme 1 – MATES staff relatability builds worker trust and facilitates engagement in the programme

Fundamental to worker trust in the MATES programme was the relatability of MATES staff. This relatability was built across a number of elements such as MATES staff most often being recruited from the industry and acting as peers from their lived experience perspectives rather than as mental health professionals, how staff integrated and became part of the jobsite and its culture, staff modelling the programme by actively participating in the MATES peer networks and the passion with which staff delivered the programme.

MATES staff were considered by participants to be peers and part of the industry. MATES staff sharing their lived experience allowed workers to feel safe to approach and engage with the MATES staff and organisation. The combination of being a peer and having lived experience bridged the gap of trust that many workers felt towards corporate and commercially offered services.

Generally, a lot of the MATES trainers and support guys, they tend to be ex‐construction people who've been influenced by suicide in some way so people can relate to that a lot better than somebody maybe from corporate coming out and telling them that there's a helpline to call if they have a problem. They're much more likely to listen to some grizzly old guy who talks about his mate dying 10 years previously or something like this.

(Construction and Commissioning Manager, Male, 43)

I love it when [Name] and other Field Officers comes, they talk at our level. They know who we are because they know the industry.

(Health, Safety, Environment, and Quality Manager, Male, 45)

The MATES staff sought to actively become part of the jobsite community through site visits and participation in site events. This approach helped MATES become a normal part of the job and culture, which in turn helped break down stigma around mental health on the site. Participants described how they would seek to extend their relationships with the Field Officers to the sites more generally. While Field Officers would come on sites to train workers and support the volunteer networks, on‐site volunteers also created opportunities such as site barbeques or toolbox talks where Field Officers could talk to the workforce directly. This was seen to create a more genuine connection between workers and the MATES organisation through the Field Officers.

Mates has always been really engaged with being out in our community and coming to sites, and it's almost part of the job now and our construction culture.

(Electrician Foreman, Male, 44)

Humans are social people we want that social emotional connection. We want [Name] (Field Officer) to get up and give us a song so we can have a laugh together. We want to connect to that person presenting the information to us. We want to empower people and that is done through that connection between the Field Officer and workers. If we just gave them a video it would not be effective at all. People become Connectors because of this connection to the MATES staff.

(Health, Safety, Environment and Quality Manager, Male, 45)

MATES Field Officers actively modelled the programme in the way they interacted on site. Staff would not just establish the programme work sites by providing training but would be an integral part of the networks of safety established on sites through their interactions with volunteers and workers generally. This person‐centred approach to engagement was highlighted by participants as giving them confidence and motivation to support and advocate for the MATES programme on their worksites. Several workers described how they modelled the support provided to them by MATES staff in the manner they provided support to other workers on site.

The strength is the constant contact with [Name], our Field Officer. He has been the main contact for me. I can reach out to him and say, “can we have a cuppa and talk about my problems as well as the site and the business.” I can always reach out to him, I think that is probably one of MATES' biggest strengths, the Field Officers who are always contactable.

(Occupational Health and Safety Coordinator, Male, 50)

The strong focus on lived experience in the MATES programme, and often the lived experience of the MATES staff, created passion and conviction in the implementation of the programme. Participants explained how this passion convinced them as workers and managers to engage in the MATES programme on their sites. Field Officers were doing more than just training and supporting workers, Field Officers were inspiring workers to participate in a joint endeavour to improve mental health and reduce suicide within their industry. Through genuine connection Field Officers effectively demonstrated the value and importance of the site accepting the MATES programme.

I reckon you'll have one interaction with them, and you'll go, ‘Jeez, we've got to do something with these guys’ because it's very powerful. You'll see if you have a genuine care around mental health, that these are the people that you'll need to create a relationship with to really strengthen your work around mental health within your organisation and for yourself as well.

(Head of Health, Safety, Environment, Quality and Training, Male, 52)

While participants valued that Field Officers were peers bringing their lived experience as construction workers, several participants also highlighted the value and importance of MATES being evidence‐based and well‐researched. Participants talked about the importance of balancing lived experience and learned expertise and saw the Field Officers as the connection point between evidence and practice. Participants expressed that they had volunteered to participate in this study because they valued the evidence base underlying the MATES programme and that they wanted to further contribute to it, but that equally it was through the passion of the Field Officers that this evidence became evidence‐based practice.

I think it's the people that you have who deliver it. It's just a genuine passion. They've generally worked in the industry, you know that it's not just someone who has come from university that knows nothing about the construction industry, and they're delivering what they know. There's nothing wrong with that, but I think if you can tie real world experience and real‐world knowledge, actual evidence‐based practice – that's kind of what it seems like. It's a bit of an amalgamation between the two.

(Electrician Forman, Male, 44)

Theme 2 – The authentic industry base of the programme enhances worker trust and engagement

The MATES organisation and programme's positionality within the construction industry was also a significant factor that built workers' trust in the programme. Work in the construction industry is often itinerant with workers moving across the industry as one project completes and another starts. That the MATES programme and organisation was applied industry‐wide was seen as significant. MATES used established industry communication channels and iconography to further strengthen the programme's industry base and focus. Being a bipartisan organisation jointly controlled by employers and unions meant that workers saw MATES as above the industry politics generally, although specifically trade union support for the programme was seen as important by rank‐and‐file workers. However, it was equally important that the MATES programme connected with workers directly and not simply relying on employer and union structures in the industry.

Participants highlighted that because MATES was industry‐based, this allowed the programme to move with them from site to site. Workers perceived their connection to a particular employer or worksite as temporary, while their connection to the industry overall was ongoing across projects and employers. In this context having a programme workers could take from site to site with them, as opposed to having to engage with individual site‐ or employer‐based programmes was seen as important for participants.

I think the MATES program works fairly well because I think there's a feeling of connection there, a wider connection. I think a lot of projects to a certain point are never a project in their own right. […] I spent quite a long time in my early career working in government where the team was very closed and you were nearly removed from the outside world, but a lot of these construction sites now, people can be working on my site today and then in two months' time they'll be working on another.

(Safety and Logistics Manager, Male, 42)

In the context of the itinerant nature of the industry, having an industry‐wide programme such as MATES was seen as worth fighting for. Workers saw the MATES programme as their programme as construction workers and would therefore not readily accept something that was seen as imposed on them from outside the industry or even their employer.

I think it's the – yeah, the fact that it's universal – I think construction workers don't like being told what to do, or anything. So, if you had the builder say hey, this is who we're going to use, we're not using MATES in Construction, people are going to be more inclined to be like, no, fuck that, I'm not going to that, I want Mates in Construction. Do you know what I mean?

(Tiler, Male, 32)

Participants highlighted how MATES is using existing construction culture and traditions to promote mental health and suicide prevention in the workplace. Construction workers often customise their hardhat with stickers communicating which sites they have worked on, their trade, the union they belong to and any specific role they may have on site such as first aid officer, union delegate or safety committee member. MATES using coloured hardhat stickers to communicate worker roles in the programme was seen as consistent with this tradition. Workers on site often decorate the workplace with trade union flags, claiming the space as a unionised workplace. Participants explained how the annual MATES “fly the flag day” brought these symbols of looking after each other together and served to reinforce the programme.

Construction is my life and MATES is everywhere on our job sites. You know what I mean it's on hard hats, it's everywhere and it's a lot more relatable I suppose compared with… If I were to do something with Beyond Blue or – I actually did the mental health first aid training probably about a month ago now and I just didn't find it the same.

(Union Delegate, Male, 36)

You've got all of the flags up. On one wall, there's the three years of MATES in Construction Fly the Flag. On the opposite walkway, all the union flags are up there. You see the flags all the time, so I guess it's in your face.

(Electrician, Male, 43)

Relationships between unions and employers in the construction industry can often be acrimonious with frequent confrontations. In this environment all issues can be contested with each party seeking to get an advantage over the other. Participants highlighted that for MATES this was different. There was a genuine industry consensus that all parties needed to work together to address suicide rates and to improve mental health in the industry. Several participants highlighted that they felt it was important to demonstrate that unions and employers could work together on big and important industry matters, and this highlighted the importance of the program.

I think one of the good things is it's union if you like ‐ it's union supported, and company supported. Most companies don't see it as a bad thing having suicide prevention, and obviously, most unions wouldn't see it as a bad thing either. It's the kind of thing everyone can agree on from my experience.

(Construction and Commissioning Manager, Male, 43)

Overt union support for the MATES programme was seen by participants as significant in workers trusting and supporting the programme. The unions could convey a message of quality and particularly safety in engaging with the MATES programme. The precarious nature of employment in the industry means that workers genuinely would be reluctant to express weakness as this could prejudice further employment and, in this context, having union approval was significant in building trust in the programme.

The union have a big part, I think, and because we're all in the union and we know about it, I think we all get more involved in it, because it's not like it's like, yep, this is MATES in Construction, and it's been going this long. They support MATES in Construction and promote it, so it's not like they just let things come into the industry if it is not good.

(Site Union Delegate, Female, 54)

Participants pointed out that it was often the union‐appointed delegate or safety representative that was driving the implementation of the MATES programme on their sites. Many of these delegates and safety representatives were not only supporting the programme but actively participating as Connectors and ASIST workers as well. For union delegates, being a MATES volunteer was seen as a natural extension of their role in caring for their members. As volunteers in the MATES programme these delegates would often be the champions for the MATES programme on site, organising and reminding project managers of the importance of implementing the MATES programme.

The Union is definitely involved in MATES. I know a lot of our union delegates they all support MATES. I'm pretty sure most of them are Connectors or ASIST workers. They always wear their union sticker on their helmet with their MATES Sticker. I know that a lot of the union posters will have the MATES logo attached to it.

(Engineering Apprentice, Male, 32)

## 4 Discussion

Various aspects of the relationship between the MATES organisation and volunteers came to light as key drivers of the widespread uptake and engagement with the MATES programme. This study found that workers saw MATES staff as peers, part of their worksite and their suicide prevention network. Participants also pointed to the passion MATES staff brought to their job as significant in building trusting relationships. The positionality of the MATES organisation and programme within the construction industry was also important to workers' trust and acceptance. The MATES programme being industry‐wide, across an industry where workers identify stronger with their industry than their jobsite or employer [21], was another important factor. MATES using existing and accepted channels of communications and symbolism was also significant as was the bipartisan support for MATES from employers and unions. Workers also highlighted the support of the unions as central to their trust and engagement with the programme.

MATES has a published programme logic map documenting presumptions and expected relationships between inputs, outputs and outcomes [28]. The programme logic map highlights a presumed relationship between an activity of industry workforce development and support targeting MATES staff, and an expected short‐term outcome of ‘*MATES staff feeling supported and satisfied’.* This short‐term outcome in turn is presumed to lead to a medium‐term outcome of ‘*Industry see MATES staff as honest, reliable, proactive and relationship based*’. The programme logic hypothesises that this industry view of MATES staff will positively impact outcomes such as ‘*improved helping behaviours*’ [28]. This is consistent with the findings of this study as well as previous studies that have demonstrated a link between MATES and increased helping behaviours [19, 22, 23, 29],

The findings in this study highlighted the significance of the MATES programme being seen as understanding and being part of the construction industry. Previous research has pointed to workers strong social identity and pride as construction workers as motivators for engaging with the programme [21], in this study workers highlighted the importance of MATES being created by the construction industry and for the construction industry as significant for their trust in the programme and organisation. This study like previous research pointed to the strong sense of ownership felt by workers in the MATES programme [29].

MATES using existing communication channels such as safety and union structures and iconography such the use of hard hat stickers, posters and flags to promote the programme was found to be important. It was also important that the programme was delivered by MATES staff with passion often informed by lived experience. In short, MATES staff were seen by workers as highly relatable. These findings were also consistent with the finding in a previous study pointing to the importance of ‘*Visibility, Camaraderie and Passion’* in the delivery of the MATES programme [29].

## 5 Limitations and Future Research

This study focused on the importance of the relationship between volunteers and the MATES organisation, programme, and staff and therefore only engaged with individuals who self‐identified as having a relationship with the MATES organisation, programme, or staff. This means that the study did not capture and include the experiences of workers who chose not to have a relationship with MATES. The study relied on volunteer participation which could lead to self‐selection bias in the sample. Further the study was limited to the state of Queensland where the MATES programme has been implemented for the longest time with the greatest reach within the industry; thus, the study's findings may have less application to jurisdictions with less programme penetration, or to Mining, Energy or Manufacturing where the programme has been more recently initiated. Further research in other jurisdictions and industries, as well as research seeking to understand whether the MATES positionality in the industry might also be a barrier to engagement, is recommended.

## 6 Conclusion

This study presented evidence that the relatability of MATES staff and the industry base of the MATES organisation and programme were seen as important reasons for workers in the construction industry to engage with and participate in the MATES programme. Combined with previous research highlighting the importance of social identity, mateship and lived experience in the Queensland construction industry this study may have wider application in preventive health promotion in other communities.

## Author Contributions

**Jorgen Gullestrup:** conceptualisation, methodology, formal analysis, investigation, data curation, writing – original draft, writing – review and editing. **Samantha Thomas:** conceptualisation, methodology, formal analysis, validation, writing – review and editing, supervision. **Tania King:** conceptualisation, validation, writing – review and editing, supervision. **Anthony D. LaMontagne:** conceptualisation, validation, writing – review and editing, supervision, project administration, funding acquisition.

## Ethics Statement

The study was approved by the Deakin University Human Research Ethics Committee (2021‐406).

## Conflicts of Interest

Jorgen Gullestrup reports a relationship with MATES in Construction Australia Limited that includes employment and funding grants. Jorgen Gullestrup has been supported by an Allison Milner Memorial PhD Scholarship while conducting this study. Anthony LaMontagne reports financial support was provided by National Health and Medical Research Council (grant number #MRF1199972). Tania King reports financial support was provided by Australian Research Council. Since completion of this study Jorgen Gullestrup has taken a position as CEO of MATES in Construction. Anthony LaMontagne is the unpaid Academic Director of MATES in Construction and Tania King is an unpaid member of the MATES in Construction Research Reference Committee.

## Supporting information


**Supporting Material 1**.

**Supporting Material 2**.
