# Supplementary material for: The Influence of Relationships on Engagement in an Australian Construction Industry Suicide Prevention Programme
Source: Am J Ind Med. 2025 Oct 31;69(1):24–31. doi: 10.1002/ajim.70034 (PMC12701561; doi:10.1002/ajim.70034)
Supplement: Supplementary file 1 — Supporting Material 1. [file AJIM-69-24-s002.docx]

Supplementary Material 1

**Interview schedule: Participants in the MATES program**

**Setting:**

- Ensure the participant has provided a signed consent form.
- Ensure the setting provides adequate privacy and it is a setting the participant feels comfortable with.
- The questions below are prompts – if a question has been answered in an earlier answer it should not be asked again.

**Introduction:**

- Provide participants context and explanation about the research project
- Ensure the participant has understood the Plain English Statement
- Inform the participant that the interview will be either video or audio recorded
- Explain in particular that the participant has the right to stop or pause the interview

(Q: are Questions asked directly of the interviewee, while “Prompts” are asked if relevant based on the answer given by the interviewee)

**About you:**

**Q:** How long have you been in the Construction industry?

**Q:** What is the highest completed formal education or training?

**Q:** What is your job title?

**Q:** Will you describe yourself as being First Nations Australian, belonging to a Culturally or Linguistically Diverse Community, identifying as LGBTIQ+ or any other identity that is significant to you?

**Q:** How would you describe your worksite:

- - Size – Very large, large, medium, small or micro?
  - Type - Commercial Construction, Residential Construction, Housing Construction, Civil Construction, Engineering Construction, Maintenance Work, Renovation Work, or Service work.

**About the MATES program:**

**Q:** How do you understand the MATES program and why did you decide to be part of the MATES program as a (Connector/ASIST worker as appropriate)?

**Prompts:**

- How would you explain the MATES program to someone who knew nothing about it?
- What is unique about the MATES program?
- What makes the MATES program suitable for the Construction industry?
- What made you become active in the program?
- What did you volunteer for this interview?

**About Mateship:**

**Q:** What can you tell me about Mateship in the construction industry?

**Prompts:**

- What does mateship mean to you?
- It there a special type of mateship in the construction industry?
- Why do you think so?

**About Supporting your mate:**

**Q:** How have you seen the MATES or other programs in action on your site or life generally?

**Prompts:**

- Have you ever noticed a workmate struggling?
- What did you do?
- What was helpful to you and what was not so helpful?

**About mental health and suicide prevention in your workplace:**

**Q:** How have you seen mental health and suicide prevention being supported in your workplace?

**Prompts:**

- How have you seen the MATES program work on your site?
- Apart from training in the MATES program are you doing any other activities?
- Do you find these are important and why?
- What other things is your site doing to promote mentally healthier workplaces?
- the MATES program has the objective that “MATES is no longer a program run on sites, but a way the industry does business” what would that mean to you?
- Is there something your worksite is not doing that you feel they should be doing?

**About a mentally healthier construction industry:**

**Q:** What do you think we can do as an industry to improve mental health and suicide prevention?

**Prompts:**

- Who should take the lead?
- Do you think we can change?
- What do you think has changed?

**About Help Seeking:**

**Q:** This is a question about your own lived experience. You do not have to answer it if you do not feel like it. If distress is defined as “An emotional state in which individuals feel that they are not in control, overwhelmed, or unable to cope” can you think of a time you felt distressed and, if you can, what helped you?

**Prompts:**

- Who did you talk to about it (a mate, colleague, family, health professional)?
- Did you call a help line?
- What did you find most useful for you?
- Did you talk to your employer about it?

**Finishing off:**

- Thank the participant for the interview.
- Explain that a transcript will be e-mailed to them and that they can withdraw from the study up to one week after having received a copy of the transcript.
- Explain that the will received an interim report once completed.

**Final Questions:**

**Q:** If you were to give one bit of advice to MATES in Construction, what would it be?

**Q:** Have you got anything else to add?
