# Supplementary material for: The Influence of Relationships on Engagement in an Australian Construction Industry Suicide Prevention Programme
Source: Am J Ind Med. 2025 Oct 31;69(1):24–31. doi: 10.1002/ajim.70034 (PMC12701561; doi:10.1002/ajim.70034)
Supplement: Supplementary file 2 — Supporting Material 2. [file AJIM-69-24-s001.docx]

Supplementary Materials 2

**Table of Quotes**

| **Theme 1 – Workers build trust in the MATES program through the relatability of MATES staff** | | |
| --- | --- | --- |
| MATES as peers – workers relate to MATES staff as construction workers | *I connect with Mates in Construction because they're like me and they come from where I come from. (Steel Fixer, Male, 42)*  *A lot of the guys who work at MATES are ex tradesmen or ex been in the industry, so you know their faces and it sort of helps build that – keep that connection I guess. (Plumber, Male, 29)*  *I think the fact that they're willing to come to site and go through if you like real world examples and share their own experiences and they're also familiar with the construction background, more effective with the team on the ground than if you had somebody from HR or something come up. (Construction and Commissioning Manager, Male, 43)*  *They don’t want a person removed from our industry to tell them how it is or isn’t in our industry. I think that’s one of the critical successes of this program. It’s been developed for people in the industry by people in industry. (Health, Safety, Environment and Quality Manager, Male, 56)* |  |
| MATES staff are integrated into the job and culture | *And then Mates in Construction come to us. So, it was sort of my way okay they've come to us so yeah I’ll join them sort of thing. So, if that makes sense. (Carpenter, Male, 35)*  *I guess because Mates was probably – was really the first, I think, company to really engage with it, but also I think because it’s reaching out and doing stuff on site, it’s not necessarily an external thing. Like, it’s kind of become included in the, you know, workplace culture and all those sorts of things. (Electrical Foreman, Male, 44)*  *For me Mates is a program that opens up the conversation about mental health, suicide in a way that it’s a supportive but informal sort of – so it takes it more as a compatriot style. (Site Manager, Male, 40)* |  |
| MATES staff model the program in the way they interact with workers | *But the few people that really helped me and spent time with me were from MATES in Construction and they kept connecting with me to make sure I was okay. I just thought that was so awesome. It really sort of cemented everything that I’ve experienced before with MATES and how they assist and help. My journey started with MATES a long time ago. (Head of Health, Safety, Environment, Quality and Training, Male, 52)*  *Mates in Construction I spoke to a Maori lady and she was beautiful. And yeah I spoke to a few other people on the phone. Couple of boys and because they're from the building industry they had jobs just like us. You know what I mean? So, they tend to know – be more like us and talk more like us. And make people feel comfortable. (Steel Fixer, Male, 42)*  *The field officers are also another support network that usually you can ring, and they can come to your location. (Health, Safety, Environment and Quality Manager, Male, 56)*  *A huge strength of the MATES guys is that they care about what they are doing, and it obviously clear and evident. They don’t just turn up, they prepared, and they follow up. For someone like me that is really easy to identify. (Health, Safety, Environment and Quality Manager, Male, 45)* |  |
| The MATES staff passion for the program is contagious | *I think it’s – like, other stuff that companies would put on, it just seemed like they were kind of ticking a box more for them, versus Mates I feel is really – they’re doing it for – or it’s done for the workers, you know, the people on the ground. It’s not a KPI or something that feels a little forced, you know? (Electrical Foreman, Male, 44)*  *I suppose just the initial GAT training, because the person we have on board delivers that very well and they’re very passionate about it and it’s very real and it’s coming from their heart and probably in some instances through personal experiences and it’s a strong message and once you’ve finished the GAT training, you think, “I’ve got to keep going with this.” (Head of Health, Safety, Environment, Quality and Training, Male, 52)* |  |
| **Theme 2 – Workers finds the MATES program credible because they see it as an integral part of their industry** | | |
| The MATES program is universal, and industry based | *I think MATES is one of the things you can take from site to site, and you can share your experiences with and you can talk to people (Safety/Logistics Manager, Male, 42)*  *Yeah, you're not just some random person that's a jack of all trades kind of thing, you know what I mean, jack of all trades, master of none. The MATES in construction, they're specifically for construction, I think guys relate to that I think (Carpenter, Male, 35)*  *You know, it’s so important, Mates is so important. It’s a symbolisation of our industry. (Plumber/Operator, Male, 52)*  *It’s people in the industry, educated in the industry, by people that have been in the industry, and understand industry, and that’s how the program was put together, and that’s how I think I would best explain it. (Field Officer, Male, 53)*  *That it’s industry-specific, that they are designed for our industry, and they understand the high-risk industry that we work in on a daily basis and the pressures involved, obviously. (Dogman, Male, 51)* |  |
| Using traditional communication channels and iconography | *But it is real, but that is why it resonates because it is an extension of the conversations we are already having on site. (Health, Safety, Environment and Quality Manager, Male, 45)*  *So, it’s actually using existing relationships but just in a different way and I think that was probably the most powerful thing for me in terms of how it broke down the barriers and had resonation with a workforce (Engineering Manager, Male, 55)*  *That was predominantly on EBA sites, so Union sites. I saw they had MATES in Construction banners here and there. At the time I didn't really know what it was about, because you see the banner, and so you like to know what it is about. That's when I first noticed it. (Engineering Apprentice, Male, 32)*  *You see more and more people with the MATES in Construction stickers on their hard hats and then you connect it with the MATES training (Plumber, Male, 29)*  *Well, I think in order to set things up right to advertise we are a MATES site, to have the posters up, having them up in the induction rooms so they know we are that sort of site. Having it in your daily pre-starts. (Senior Construction Manager, Male, 44)* |  |
| MATES is a bi-partisan initiative | *Everyone can come together, and it goes beyond just your company, it goes beyond just your trade, even your trade union. All the unions onsite, no matter who you are, electrician, plasterer, plumber, brick layer, everyone treats each other on the same level and then the MATES things come into that. (Plumber, Male, 29)*  *I’ve noticed any site it’s been on; it’s always promoted across the board. No one questions who funds Mates, because its industry funded. No one questions the private parts of it, the confidentiality. No one has ever questioned that, because it come from the grassroots (Electrician, Male, 43)*  *It wouldn’t matter if you were talking with a Master Builders, or you were talking with a project director, or you were talking with the cleaner at the job, they all understand what Mates in Construction is. (Plumber/Operator, Male, 52)*  *Yeah 100%, that’s one thing about MATES is it’s not just for the workers right, I’m sure that supervisors and foremen and stuff utilise it as well. They go through the similar struggles that we do. (Union Delegate, Male, 36)* |  |
| The Union support for MATES is important | *I think a lot of it is to do with the union as well, the fact that a lot of the guys, the majority of people are in the union, not everybody obviously, and the union does promote family-orientated values – and MATES in Construction as being mates. But family, I think, is a big thing too. A lot of people tend to – if they see signs of getting a little bit bullied or harassed and people won’t just stand back and let it happen. They’ll jump in and pull it up. I think that’s a big thing that the union has been behind, and I think that’s why MATES in Construction and the union are very good together. (Health and Safety Representative, Male, 57)*  *I think the fact that Mates in Construction has such a massive backing from CFMEU shows that that’s obviously the perfect fit for our industry. (Tiler, Male, 32)*  *I think today, with the fact that the union have, you know, done a lot with Mates in Construction as well, it’s changed a lot of attitudes, where it’s okay to talk about it. (Site Delegate, Female, 54)*  *Like I said, all three unions support and work with MATES in Construction. So again, it’s all that unity and that team stuff. (Electrician, Male 43)* |  |
